# Mitochondrial dysregulation and muscle disuse atrophy

**DOI:** 10.12688/f1000research.19139.1

**Published:** 2019-09-11

**Authors:** Li Li Ji, Dongwook Yeo

**Affiliations:** 1 The Laboratory of Physiological Hygiene and Exercise Science, University of Minnesota Twin Cities, Minneapolis, MN, 55455, USA

**Keywords:** Atrophy, Mitochondria, Muscle, PGC-1α, Redox Signaling

## Abstract

It is well established that mitochondria play a critical role in the metabolic and physiological adaptation of skeletal muscle to enhanced contractile activity. Several redox-sensitive signaling pathways such as PGC-1α, AMPK, IGF/Akt/mTOR, SIRT, NFκB, and FoxO are involved with extensive crosstalk to regulate vital cellular functions such as mitochondrial biogenesis, mitochondrial fusion and fission dynamics, autophagy/mitophagy, and apoptosis under altered demand and stress. However, when muscles cease contraction, such as during immobilization and denervation, mitochondria undergo a series of detrimental changes characterized by downregulation of PGC-1α and antioxidant defense, increased ROS generation, activated FoxO, NFκB, and inflammation, enhanced ubiquitination, and finally mitophagy and apoptotic cascades. The phenotypic outcome of the discord of mitochondrial homeostasis is elevated proteolysis and muscle atrophy. The demonstration that PGC-1α overexpression via transgene or *in vivo* DNA transfection can restore mitochondrial homeostasis and reverse myocyte atrophy supports the “mitostasis theory of muscle atrophy”.

## Introduction

In mammalian skeletal muscle, the role of mitochondria in maintaining proper oxygen consumption and ATP production, thereby fulfilling metabolic and contractile functions, is well known ^1^. Over the past several decades, overwhelming evidence has revealed new cellular roles for this organelle, such as to regulate redox homeostasis between ROS and antioxidant defense as well as to control apoptosis ^2– 4^. Furthermore, the mitochondrion itself undergoes morphological, structural, and genetic alterations to accommodate the above-mentioned changes, mainly via mitochondrial biogenesis and turnover ^5– 7^. The latter is controlled by mitochondrial fusion and fission dynamics, ubiquitin proteolysis, and lysosomal–autophagy (mitophagy) pathways ^8– 11^. Within the past three decades, there have been tremendous integrations of knowledge among biochemistry, molecular biology, immunology, genetics, and cancer research to elucidate the mechanisms for governing cell growth, differentiation, adaptation, and death. Among these discoveries, PGC-1α has taken the front stage ^12^. Indeed, the multiple roles of PGC-1α to control mitochondrial biogenesis and fusion–fission dynamics, its interaction with NFκB and Forkhead box class O family member proteins (FoxO), and its influences on the Sirt pathway and apoptotic cascades have dominated muscle physiology and exercise physiology ^13– 18^. Instead of providing an overview of mitochondria, this short communication will focus on the role of this organelle in regulating muscle protein degradation during disuse atrophy. Understanding the cellular mechanisms of muscle atrophy may provide insights into the development of therapeutic treatment for patients suffering from muscle wasting.

A wealth of research has demonstrated that reduction, restriction, or complete cessation of contractile activity of striated muscle due to denervation, bed rest, microgravity, and senescence can lead to the loss of muscle mass and function ^19^. The primary outcome of muscle immobilization (IM) is increased proteolysis, oxidative stress, inflammation, and functional deterioration ^20, 21^. During muscle IM, reduced stimulation from the IGF–Akt–mTOR axis leads to lower protein synthesis, and activation of the ubiquitin-proteolysis and autophagy-lysosomal pathways that enhance protein degradation. The majority of research suggests that increased proteolysis is the main reason for muscle protein loss, although diminished protein synthesis also plays a role ^20^.

Atrogin-1 and MuRF1, muscle-specific E3 ubiquitin ligases, activate protein degradation ^22, 23^ by controlling the ubiquitination and degradation of regulatory (e.g. calcineurin and MyoD) as well as structural (e.g. myosin and troponin I) proteins ^24– 27^. In particular, remobilization (RM) of a muscle that experienced a prolonged period of IM does not undo the effects of this IM immediately; rather, IM-RM has been shown to promote ROS generation, activate the NFκB pathway, and subsequently stimulate the expression of pro-inflammatory cytokines such as TNFα, IL-1β, and IL-6 as well as inflammatory myokines and cause oxidative stress ^28^.

## Critical role of mitochondria in muscle disuse atrophy

Research over the past two decades suggests that loss of mitochondrial homeostasis (mitostasis) can be a primary reason for observed muscle morphological and functional defects after an extended period of disuse ^29^. All experimental models of muscle atrophy consistently revealed prominent reduction of mitochondrial volume density. After two weeks of hindlimb IM in mice, mitochondrial density was decreased by 50% in several muscles ^30, 31^, with a further 25% loss in the third week ^30^. Considering the reduction of muscle fiber cross-section area during the corresponding time, the total reduction of mitochondrial quantity is devastating. There is some evidence that the majority of loss was the subsarcolemmal mitochondrial subpopulation ^32, 33^.

In addition to the quantity change, mitochondrial quality also severely deteriorates during muscle IM. Activities of mitochondrial metabolic enzymes such as citrate synthase (CS) and cytochrome c oxidase (COX) showed a severe reduction, accompanied by a 50% decline of ATP production rate, indicating the muscle was energy deficient ^30^. The abundance of mitochondrial DNA (in proportion to nuclear DNA) also showed a reduction. It is not entirely clear whether the defects shown in mitochondrial quantity and quality were the cause or effect of muscle atrophy; however, there is evidence that the occurrence of mitochondrial deterioration precedes the loss of muscle mass ^34^.

A skeletal muscle mitochondrion has a half-life of approximately two weeks; therefore, the decline of its volume can be caused by a decrease of biosynthesis, an acceleration of degradation, or both. Mitochondrial biogenesis is controlled primarily by PGC-1α, co-activation of which promotes the expression of Nrf-1 and Nrf-2, a key step for the gene expression of nuclear-encoded mitochondrial proteins and of Tfam, the key regulator of mitochondrial DNA (mtDNA) biosynthesis ^35^. PGC-1α mRNA and protein levels have been shown to decrease steadily during muscle IM for 1–3 weeks ^28, 30, 36, 37^, along with the reduction of Nrf-1 and Tfam ^30^. Whether or not downregulation of PGC-1α is the primary trigger for the decline of mitochondrial biogenesis still requires verification, as PGC-1α itself is also subject to transcriptional and post-translational regulation by other signaling pathways ^38^.

In an atrophying muscle, decreased mitochondrial quality and quantity is also influenced by its degradation, controlled by mitophagy, as well as the fusion and fission dynamics ^31, 39, 40^. A decline of mitochondrial inner membrane potential (Δψ _m_) may serve as the initial signal for the relocation of PINK1 to the mitochondrial membrane, which phosphorylates mitochondrial fusion protein-2 (Mfn2) as the docking point for Parkin, a ubiquitin ligase ^41^. Beclin 1, BCL2/Adenovirus E1B 19kDa Interacting Protein 3 (Bnip3), microtubule-associated protein 1 light chain 3 (LC3), and the autophagy adaptor protein p62/SQSTM1 (p62) are key players for forming the autophagosome, which engulfs mitochondria followed by lysosomal degradation ^41^. In skeletal muscle, Beclin 1 and Bnip3/nix upregulation are controlled by FoxO ^42^. Activation of this PINK1–Mfn2–Parkin axis facilitates the removal of damaged mitochondria to maintain a healthier mitochondrial pool but decreases overall mitochondrial population in the disused muscle. After two weeks of IM followed by RM, both PINK1 and Parkin expression was increased severalfold ^31, 43^. Increased mitophagy during IM is a double-edged sword; it eliminates damaged and dysfunctional mitochondria to keep a smaller but healthier mitochondrial population but sacrifices mitochondrial quantity and causes a deficit of energy production.

## Crosstalk of signaling pathways during muscle atrophy

**Figure 1.**
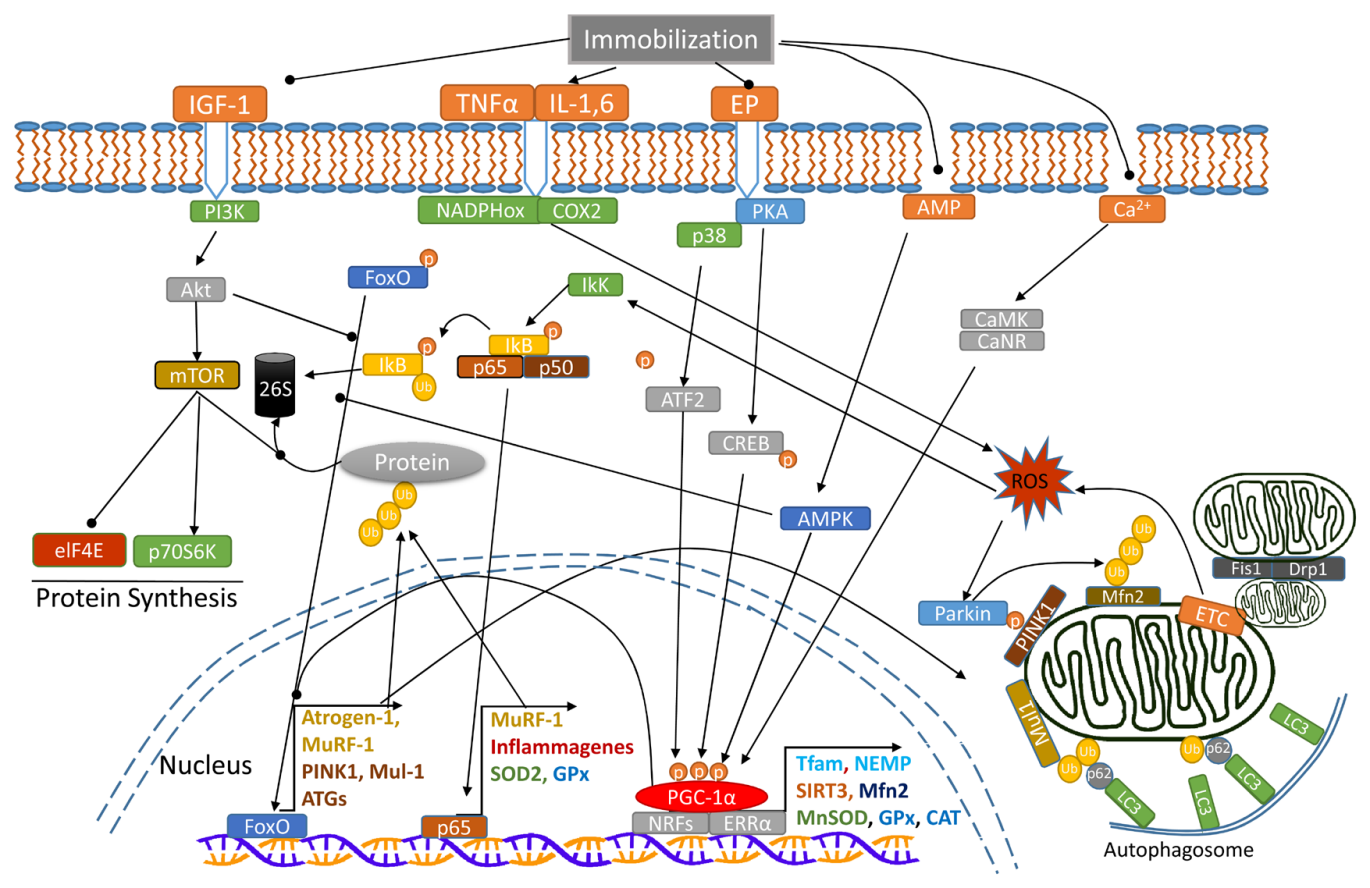
Illustration of the effects of muscle immobilization on intracellular signaling pathways causing increased ubiquitin proteolysis and mitophagy. Arrow-headed lines represent activation; dot-ended lines represent inhibition. 26S, 26 proteasome; Akt, protein kinase B; ATF2, activating transcription factor 2; ATGs, autophagy related proteins; CaMK, Ca ^2+^/calmodulin-dependent protein kinase; CaNR, calcineurin; CAT, catalase; COX2, cytochrome c oxidase 2; CREB, cyclic AMP response element-binding protein; Drp1, dynamin-related protein 1; elF4E, eukaryotic translation initiation factor 4E; EP, epinephrine; ERRα, estrogen-related receptor alpha; ETC, electron transport chain; Fis1, mitochondrial fission 1 protein; FoxO, Forkhead box class O family member proteins; GPx, glutathione peroxidase; IGF-1, insulin-like growth factor 1; IkK, IkB-kinase; IL-1,6, interleukin-1,6; LC3, microtubule-associated protein 1 light chain 3; Mfn2, mitofusin-2; MnSOD, manganese superoxide dismutase; mTOR, mammalian target of rapamycin; Mul-1, mitochondrial E3 ubiquitin ligase; MuRF-1, muscle RING-finger protein-1; NADPHox, nicotinamide adenine dinucleotide phosphate oxidase; NEMP, nuclear-encoded mitochondrial proteins; NRFs, nuclear respiratory factors; p, phosphate; p38, p38 mitogen-activated protein kinase; p50, p65, NFκB subunits; p62, sequestosome 1; p70S6K, ribosomal protein S6 kinase beta-1; PGC-1α, peroxisome proliferator-activated receptor gamma coactivator 1-alpha; PI3K, phosphatidylinositol 3-kinase; PINK1, PTEN-induced kinase 1; PKA, protein kinase A; ROS, reactive oxygen species; SIRT3, sirtuin-3; SOD2, superoxide dismutase 2; Tfam, mitochondrial transcription factor A; TNFα, tumor necrosis factor alpha; Ub, ubiquitin.

There is strong evidence that alteration of mitochondrial morphological changes due to fusion and fission protein expression affects many vital cellular functions and is critical to mitostasis ^44^. Mfn2 repression was shown to decrease the rate of pyruvate and glucose oxidation, reduce mitochondrial membrane potential (Δψ _m_), and cause a dramatic discontinuity of the mitochondrial network ^45^. Interestingly, FoxO activation during muscle atrophy promotes the expression of mitochondrial E3 ubiquitin ligase (Mul-1), thus ubiquitinating and degrading Mfn2 ^46^. Therefore, IM may promote mitochondrial fission and fragmentation partly because of the upregulation of Mul-1 and the subsequent downregulation of Mfn2 ^31^. Furthermore, Mfn1 and Mfn2 are also substrates for Parkin, suggesting increased mitophagy favors a trend of fission ^47^. Notably, Parkin’s substrates also include other important proteins such as mitochondrial Pho GTPase, membrane translocase (TOM70, TOM40 and TOM20), and voltage-dependent anion channel proteins (VDAC) ^41^. Decreased fusion and increased fission protein expression can make mitochondria more fragmented and easier to be isolated for removal by mitophagy ^48^. Figure 1 is an illustration of the interactions of various signaling pathways that regulate mitochondrial homeostasis and muscle protein degradation.

FoxO signaling plays a number of important parts in skeletal muscle plasticity; these include energy metabolism, protein degradation, and muscle regeneration, among others ^49^. In the case of muscle disuse atrophy, FoxO3 is in control of the ubiquitin–proteasome and autophagy–lysosome pathways independently. In actively contracting skeletal muscle, the PI3K–Akt–mTOR pathway phosphorylates and inactivates FoxO, thereby inhibiting excessive ubiquitin–proteolysis and mitophagy. Contraction-mediated PGC-1α signaling and mitochondrial biogenesis maintain a healthy mitochondrial turnover and keep FoxO in check via its phosphorylation ^49^. Furthermore, PGC-1α regulates intracellular redox status by reducing ROS generation (due to a healthier mitochondrial population) and upregulating antioxidant enzymes ^14, 30^. This homeostatic balance can be disrupted within the disused muscle because of mitochondrial membrane damages shown by enhanced lipid peroxidation and ubiquitination ^30^. Reduced PI3K–Akt–mTOR pathway activity during muscle IM results in FoxO3 dephosphorylation, which then leads to its nuclear sequestration and DNA binding ^49, 50^. Furthermore, activated AMPK phosphorylates serines 413 and 588 of FoxO3a, which supports its retention in the nucleus ^51^. The activation of FoxO3 increases Atrogin-1 and MuRF-1 transcriptional activity ^52, 53^. Moreover, prolonged IM can activate NFκB signaling and increase the production of pro-inflammatory cytokines and myokines such as TNF-α, IL-1β, IL-6, and MCP ^30, 54, 55^. Both TNF-α and IL-1β are known stimulators of ROS generation from the mitochondrial electron transport chain and other oxidases such as NADPH oxidase, COX-2, and lipoxygenase, thus escalating oxidative stress via a vicious cycle.

Crosstalk of catabolic signaling pathways can disrupt mitostasis and elicit catastrophic cascades toward apoptosis in disuse atrophy. During IM, PINK1–Mfn2–Parkin axis-induced mitochondrial ubiquitination, fragmentation, and mitophagic degradation leads to differential expression of Bcl2 family proteins involved in both autophagy and apoptosis ^41^. Muscle IM has been reported to increase the relative content of Bax (Bax/Bcl2 ratio), which is associated with the activation of caspase-3 ^43^. Bnip3, the pro-apoptotic protein, can be upregulated with IM when increased ROS and inflammation prevail ^31, 43^. Bax is required for Bnip3-induced loss of mitochondrial inner membrane potential (ΔΨm), which further destabilizes the mitochondrial membrane and enhances mitophagic tendency ^56^. Furthermore, Bnip3 can induce cell death through atypical apoptosis without caspase-3 and cytochrome c release ^56^. These findings suggest that mitochondrial dynamics change and mitophagy is closely related to muscle cell death. Thus, besides decreased myocyte cross-section area (sign of atrophy), myocyte number may also decrease due to apoptosis, although an unequivocal conclusion on this matter still requires more investigation.

## *In vivo* PGC-1α transfection inhibits muscle atrophy

Because of the critical role of mitostasis in the pathogenesis of muscle disuse atrophy, various experimental models have been employed to boost mitochondrial biogenesis and to inhibit proteolytic, autophagic, and apoptotic pathways, such as FoxO gene knockout ^23^, inhibition of NFκB ^57^, antioxidant intervention, and transgenic overexpression of PGC-1α ^37, 58^. Moreover, exercise has been consistently demonstrated to be a very effective way to enhance mitochondrial biogenesis mainly due to the upregulation of PGC-1α ^17, 18, 32^. These methods have been reviewed extensively in the past; therefore, the following is a brief review of the efficacy of an *in vivo* DNA transfection technique that overexpresses PGC-1α in mouse muscle to reveal the mechanism of action ^30, 59^.

PGC-1α transfection *in vivo* was shown to effectively restore PGC-1α content in the cytoplasm, nucleus, and mitochondria ^30^, and overexpression does not seem to be limited by animal age ^31, 59, 60^. As a result, mitochondrial density and mtDNA content were both elevated in the transfected TA muscle, along with higher levels of Tfam, suggesting these improvements were due in part to increased mitochondrial proliferation. Mitochondrial oxidative function showed significant enhancement demonstrated by increased CS, COXIV activity, and ATP production rate. These findings were in general agreement with data obtained in transgenic animal studies overexpressing PGC-1α ^57, 58^.

**Figure 2.**
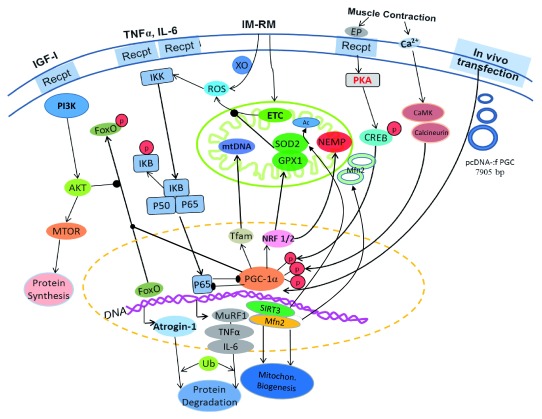
Illustration of the effects of *in vivo* PGC-1α transfection on intracellular signaling pathways that promote mitochondrial biogenesis and inhibit ubiquitin proteolysis and mitophagy. Arrow-headed lines represent activation; dot-ended lines represent inhibition. Ac, acetate; Akt, protein kinase B; CaMK, Ca ^2+^/calmodulin-dependent protein kinase; CREB, cyclic AMP response element-binding protein; PI3K, phosphatidylinositol 3-kinase; EP, epinephrine; ETC, electron transport chain; FoxO, Forkhead box class O family member proteins; GPX1, glutathione peroxidase 1; IGF-1, insulin-like growth factor 1; IKK, IkB-kinase; IL-1,6, interleukin-1,6; IM-RM, immobilization–remobilization; Mfn2, mitofusin-2; MTOR, mammalian target of rapamycin; MuRF1, muscle RING-finger protein-1; NEMP, nuclear-encoded mitochondrial proteins; NRF 1/2, nuclear respiratory factors 1/2; p, phosphate; p50, p65, NFκB subunits; pcDNA-:f PGC, PGC-1α plasmid; PGC-1α, peroxisome proliferator-activated receptor gamma coactivator 1-alpha; SIRT3, sirtuin-3; SOD2, superoxide dismutase 2; Tfam, mitochondrial transcription factor A; TNFα, tumor necrosis factor alpha; Ub, ubiquitin; XO, xanthine oxidase.

PGC-1α local transfection decreased muscle oxidative stress, such as lipid and protein oxidative damage ^30^. The protection may stem from two events: reduced ROS generation in the mitochondria and increased antioxidant defense ^30^. Enhanced mitochondrial biogenesis leads to a “younger” and healthier mitochondrial population with fewer inner membrane defects, which is a main source of ROS generation. Increased PGC-1α reduced acetylation of mitochondrial SOD (SOD2), making it more active ^30^. There is evidence that this protection is caused by PGC-1α-induced upregulation of SIRT3, a mitochondrial deacetylase. Other key mitochondrial enzymes susceptible to acetylation may also be protected by SIRT3 upregulation ^61^. PGC-1α transfection also increased muscle glutathione peroxidase (GPx) and catalase activities that control hydrogen peroxide concentration in the IM muscle ^30, 59^. Moreover, PGC-1α overexpression inhibited NFκB and the expression of IM-induced inflammatory cytokines such as TNFα, IL-1β, and IL-6, which are important negative regulators of mitochondrial homeostasis. Figure 2 summarizes the major effects of *in vivo* PGC-1α transfection on muscle mitochondrial and protein homeostasis.

IM-activated mitophagy was shown to be suppressed by PGC-1α overexpression in both young and aged mouse muscles ^31, 59^. Major players of mitophagy such as PINK1, Parkin, Mul-1, and LC3II were upregulated in mouse TA muscle after IM, but these effects were mitigated by PGC-1α transfection. Suppression of FoxO signaling probably played a critical role in this protection because it is the primary activator of mitophagy. As strong supporting evidence, mitochondrial ubiquitination level was attenuated by PGC-1α transfection. It is interesting to note that PGC-1α also inhibited Fis-1 and Drp-1 expression in the aged muscle ^59^. This finding suggests that ameliorated changes in mitochondrial dynamics by PGC-1α may attenuate mitochondrial fragmentation, a main reason for higher mitophagy rate in the IM muscle.

Recent research indicates that an interplay between PGC-1α and transcription factor EB (Tfeb) exists in skeletal muscle that regulates the biological outcome of mitochondrial biogenesis and mitophagy ^62^. Tfeb is regarded as the master regulator of lysosomal biogenesis in the autophagy process, and exercise has been shown to induce Tfeb expression ^63^. One may speculate that high levels of PGC-1α may decrease Tfeb expression, thereby attenuating mitophagy in an atrophying muscle. Interestingly, several recent studies revealed an opposite result. For example, Vainshtein *et al*. ^64^ showed that Tfeb protein level was elevated in denervation-induced muscle atrophy, whereas transcriptional PGC-1α overexpression increased muscle Tfeb and most mitophagy-related proteins. Thus, whether or not Tfeb played a role in accounting for the decreased mitophagy in IM muscle is unclear and requires further investigation.

## Conclusion

Skeletal muscle atrophy caused by IM represents a pathophysiological disorder characterized by excessive proteolysis and associated functional defects. Overwhelming evidence suggests that loss of mitochondrial homeostasis plays a critical role wherein decreased mitochondrial biogenesis, disrupted fusion–fission dynamics, and increased ROS generation and inflammation lead to enhanced mitophagy and eventually apoptosis. PGC-1α is a key transfection cofactor that crosstalks with all major signaling pathways to protect against catabolic signals. Muscle disuse atrophy can be caused by other experimental conditions such as denervation, bedrest, hindlimb unloading, and microgravity. They may share similar molecular mechanisms that cause IM-induced atrophy but may be governed by separate and unique etiological events, which are beyond this short review. It should also be mentioned that the conclusions of this review are primarily drawn from animal, mainly rodent, studies. The outcome and mechanism of human muscle disuse atrophy could be different. Nevertheless, all research to this date emphasizes the role of mitochondria as the most important organelle that controls the progress of muscle disuse atrophy, thus providing a potential target for intervention and treatment.
